# Tocotrienol is a cardioprotective agent against ageing-associated cardiovascular disease and its associated morbidities

**DOI:** 10.1186/s12986-018-0244-4

**Published:** 2018-01-19

**Authors:** Nardev Ramanathan, Esther Tan, Li Jun Loh, Boon Seng Soh, Wei Ney Yap

**Affiliations:** 1 Department of R&D, Davos Life Science Pte Ltd, 3 Biopolis Drive, #04-19, Davos, 138623 Singapore; 2 Department of R&D, Level 8, Menara KLK 1,Jalan Pju 7/6,Mutiara Damansara, 47810, 47800 Petaling Jaya, Selangor Malaysia; 3 Disease Modeling and Therapeutics Laboratory, Institute of Molecular and Cell Biology, 61 Biopolis Drive Proteos, Singapore, 138673 Singapore; 4 Department of Biological Sciences, National University of Singapore, Singapore, 117543 Singapore; 5 Key Laboratory for Major Obstetric Diseases of Guangdong Province, The Third Affiliated Hospital of Guangzhou Medical University, Guangzhou, 510150 China

**Keywords:** Cardiovascular disease (CVD), Ageing, Tocotrienols, Tocotrienol-rich fraction (TRF), α-tocopherol, Inflammation, Inflammaging, Oxidative stress, Atherosclerosis, Reactive oxygen species (ROS)

## Abstract

Ageing is a nonmodifiable risk factor that is linked to increased likelihood of cardiovascular morbidities. Whilst many pharmacological interventions currently exist to treat many of these disorders such as statins for hypercholesterolemia or beta-blockers for hypertension, the elderly appear to present a greater likelihood of suffering non-related side effects such as increased risk of developing new onset type 2 diabetes (NODM). In some cases, lower efficacy in the elderly have also been reported. Alternative forms of treatment have been sought to address these issues, and there has been a growing interest in looking at herbal remedies or plant-based natural compounds. Oxidative stress and inflammation are implicated in the manifestation of ageing-related cardiovascular disease. Thus, it is natural that a compound that possesses both antioxidative and anti-inflammatory bioactivities would be considered. This review article examines the potential of tocotrienols, a class of Vitamin E compounds with proven superior antioxidative and anti-inflammatory activity compared to tocopherols (the other class of Vitamin E compounds), in ameliorating ageing-related cardiovascular diseases and its associated morbidities. In particular, the potential of tocotrienols in improving inflammaging, dyslipidemia and mitochondrial dysfunction in ageing-related cardiovascular diseases are discussed.

## Background

Cardiovascular disease (CVD) is the number one cause of mortality globally according to the World Health Organisation (WHO). By 2030, approximately 30 million individuals are expected to die from CVD every year [1]. While recent changes in dietary habits and lifestyle are often discussed as a major contributing factor for this phenomenon, ageing presents another fertile ground for lowering the threshold required for the manifestation of the disease.

While the exact mechanics of ageing is still subject to active research, a couple of theories currently prevail as the leading theories that might explain, at least in part, the pathophysiological aspects of ageing. These are the free radical theory and the inflammaging theories of ageing [2, 3]. The former purports that with ageing, even in healthy ageing, there is a higher risk of oxidative stress build-up within the mitochondria, which eventually leads to a vicious cycle that leads to further damaged mitochondria and increased free radicals [2]. This increased oxidative stress is a risk factor for the development of chronic diseases such as cancer and diabetes. Inflammaging, on the other hand, refers to the chronic low grade inflammation that persists and leads to chronic disease [4]. These are also discussed in more detail throughout the review.

From a cardiovascular health point of view, ageing can thus be viewed as a combination of deteriorating cardiovascular protection mechanisms and a concomitant increase in disease processes that greatly increases the development of heart failure. Half of all heart failure diagnoses and 9 out of 10 of all heart failure deaths occur in the segment of the population over age 70 [5]. Hence, it would not be an overstatement to say that heart failure is a major risk for the elderly.

Many pharmaceutical interventions exist such as statins and beta blockers, but, as will be described later, many of these can have adverse events, especially in the elderly, whose physiologies are much more susceptible to certain types of pharmaceutical intervention [6–8]. There has recently been a growing interest in exploring treatment using naturally occurring compounds. While there has been scepticism about the efficacy of these compounds [9–11], in often cases, the irregular efficacies reported are due to the complex and inconsistent preparation of these herbal remedies [12]. With an increased scrutiny on manufacturing and regulatory practices in the nutraceutical industry worldwide, there has been a growing acceptance of nutrition in both consumer healthcare space and clinical settings as a bona fide interventional strategy in combating all types of human disease [12–14].

In particular, nutritional strategies have been reported to effectively lower the risk of the toxicity caused by reactive oxygen species (ROS) and hence improve in vivo antioxidant status which is important in preventing not just cardiovascular disease but many other human diseases such as cancer, neurodegeneration and diabetes as well [15, 16].

## Tocotrienol

**Fig. 1:**
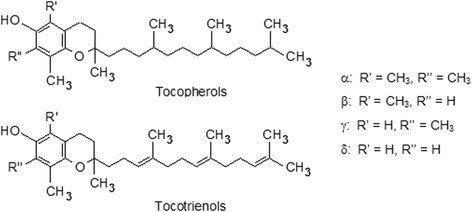
Structural configuration of tocotrienols and tocopherols. Adapted from http://lipidlibrary.aocs.org/Analysis/content.cfm?ItemNumber=40389

The Vitamin E family consists of tocopherols and tocotrienols, with each of the two groups consisting of 4 different homologues each (α, β, γ and δ). Structurally, tocopherols and tocotrienols share the identical chromanol head and only differ by the degree of saturation of hydrophobic tridecyl chain. Tocopherols have saturated phytyl tails whereas tocotrienols have unsaturated isoprenoid side chain with three double bonds. The isomers differ from each other by the attachment of different R-groups on the chromanol head (Fig. 1) [17].

Vitamin E has traditionally been associated with tocopherols only. Tocopherols were discovered nearly a century ago, in 1922 by Evans and Bishop [18] and enjoyed a dominant and lengthy period in the limelight. Tocopherols are found in most vegetable oils, nuts, seeds and whole grains [19].

Tocotrienols on the other hand is a more recent discovery. Qureshi and colleagues first differentiated tocotrienols and its properties from tocopherols in 1986 [20]. In the years that followed, several groups identified palm (*Elaeis guineensis)* oil as a rich source of tocotrienols Approximately 75% of the Vitamin E from palm oil consists of tocotrienols [21]. Another source of tocotrienols is found in the bright red seeds of *Bixa orellana*, from the achiote tree, a plant native to South America. Its seeds contain a unique composition of 90% δ-tocotrienol and 10% γ-tocotrienol, without any tocopherols in them [17, 22, 23].

The discovery of more Vitamin E compounds may seem as merely an academic pursuit, especially given the highly similar structural makeup of tocotrienols and tocopherols (Fig. 1). However, the discovery that tocotrienols may possess as much 40–60 times more antioxidative activity than tocopherols captured the attention of many [24, 25]. Further studies also suggested that tocotrienols also exhibit potent anti-inflammatory properties, a property that is only very weakly, if at all, observed in tocopherols [26–28]. In particular, tocotrienols have been shown to inhibit pathways that involve nuclear factor κB (NF-κB) [29], Signal transducers and activators 3 (STAT3) [30] and cyclo-oxygenase 2 (COX-2) [31], which are critical pathways that trigger pathological inflammatory responses.

As it became clearer that tocotrienols could be a much more bioactive form of Vitamin E compared to tocopherols, much interest in the therapeutic applications of tocotrienols arose. Chandan Sen and colleagues, for example, published many seminal papers on the potency of tocotrienols in being neuroprotective against stroke [32–35]. We and others have shown the potency of the gamma/delta tocotrienol isomers in ameliorating cardiovascular and metabolic disease [36–38].

Palm tocotrienol-rich fraction (TRF) has been granted the GRAS (Generally Regarded as Safe) status by the US FDA in 2010, indicating that these compounds are safe for human consumption [39]. Clinical trials done in humans at approximately 50–400 mg/day (equivalent to up to 6.7 mg/kg for a 60 kg human) for periods of 2 weeks to 18 months have not been reported to cause adverse effects [17], even in the elderly [40]. Tocotrienols has been shown to be cardioprotective in numerous cell culture, animal model and human studies [17, 38, 41, 42] (Table 1). With the increase in life expectancy of the world population in general, driven by improvements in medical science and healthcare technologies, leading causes of disability-adjusted life years (DALYs) predicted by one study was aging related heart disease [43]. It is thus critical to find ways to remedy cardiovascular ageing with safe and effective interventional strategies. In this review article, we discuss the potential of tocotrienols in combating ageing related cardiovascular diseases.

**Table 1:** Summary table listing the clinical studies with tocotrienols in cardiovascular diseases during the period of 1991–2011

S/N	Journal title	References
1	Lowering of serum cholesterol in hypercholesterolemic humans by tocotrienols (palmvitee)	Qureshi A.A. et al. (1991). *American Journal of Clinical Nutrition*. [79]
2	Effect of a palm-oil--vitamin E concentrate on the serum and lipoprotein lipids in humans^1,3^	Tan D.T.S. et al. (1991). *American Journal of Clinical Nutrition.* [17]
3	Differential serum responses of tocopherols and tocotrienols during vitamin supplementation in hypercholesterolaemic individuals without change in coronary risk factors	Wahlqvist M.L. et al. (1992). *Nutr Res.* [84]
4	Antioxidant effects of tocotrienols in patients with hyperlipidemia and carotid stenosis	Tomeo A.C. et al. (1995). *Lipids.*
5	Novel tocotrienols of rice bran modulate cardiovascular disease risk parameters of hypercholesterolemic humans.	Qureshi A.A. et al. (1997). *The Journal of Nutritional Biochemistry.*
6	Synergistic effect of tocotrienol-rich fraction (TRF25) of rice bran and lovastatin on lipid parameters in hypercholesterolemic humans	Qureshi A.A. et al. (2001). *Journal of Nutritional Biochemistry* [79]
7	Dose-dependent suppression of serum cholesterol by tocotrienol-rich fraction (TRF25) of rice bran in hypercholesterolemic humans	Qureshi A.A. et al. (2002). *Atherosclerosis*.
8	Dose Dependent Elevation of Plasma Tocotrienol Levels and Its Effect on Aterial Compliance, Plasma Total Antioxidant Status, and Lipid Profile in Healthy Humans Supplemented with Tocotrienols Rich Vitamin E	Rasool A.H.G. et al. (2006) *Journal of Nutritional Science and Vitaminology*.
9	Gamma Delta Tocotrienols Reduce Hepatic Triglyceride Synthesis and VLDL Secretion	Zaiden N. et al. (2010). *Journal of Atherosclerosis and Thrombosis*.
10	Effect of Mixed-Tocotrienols in Hypercholesterolemic Subjects	Yuen K.H. et al. (2011). *Functional Foods in Health and Disease*. [96]

## Cardiovascular changes that occur with ageing

Most of the major cardiovascular changes that take place during cardiovascular ageing could be categorised into one or more of the following:

A. Structural changes
B. Functional changes
C. Indirect changes
D. Decreased capacity to handle oxidative stress

### A) Structural changes

The most evident structural cardiac change during ageing is the increase in myocardial thickness. An increase in cardiomyocyte size accounts for left ventricular hypertrophy (LVH) during ageing [5]. It is also worth noting that while ventricular walls thicken as a result of increased cardiomyocyte size, there is actually a decrease in cardiomyocyte cell number, probably due to apoptosis [44]. Since the heart comprises cells that have little regeneration capacity, regardless of the scale of cardiomyocyte loss, the contractile efficiency would be affected [45].

An additional trigger for ageing LVH is the dilation of the aortic roots as it increases the inertial load in which the heart must pump [44, 46]. Structural change in combination with myocardial thickening, dilation of aortic roots also results in a change in overall heart structure. The aorta dilates rightward, extending into the cavity of the left ventricle, causing a shift from elliptical to a rounded geometry and thus subjecting it to a higher wall stress [44, 47]. Overall contractile efficiency will be affected by these structural changes and the widely-used treatment thus far is with anti-hypertensive drugs such as angiotensin receptor blockers (ARBs), β-adrenoceptor antagonists, *α*-adrenoceptor antagonists, calcium antagonists, angiotensin converting enzyme (ACE) inhibitors and diuretics [48, 49].

Antioxidant therapy has been proposed as a promising intervention that may prevent heart failure [50]. Vitamin C and E have both been studied, either individually or in combination, in improving arterial compliance and arterial stiffness [51], which are intricately linked to ventricular hypertrophy [52]. Rasool et al., found that administration of 100 and 200 mg/day of tocotrienols improved arterial compliance (as assessed by carotid femoral pulse wave velocity (PWV) and augmentation index (AI)) in healthy men [53]. Rasool et al. also used tocopherols in treating arterial stiffness in postmenopausal women but found that tocopherols did not show a significant improvement [54]. More studies will be needed with tocotrienols in arterial stiffness and compliance, particularly studies that compare the effects of tocotrienols and tocopherols directly, but these early data indicate that tocotrienols may be a better candidate than tocopherols in improving arterial compliance and stiffness.

As arterial compliance and stiffness are major complications reported in elderly cardiovascular patients, tocotrienols may present a suitable intervention strategy to achieve better clinical outcomes.

### B) Functional changes- inflammaging and immunocardiology

The phenomenon, where low grade inflammation in the absence of other significant medical conditions, as a chronic process that eventually presents a risk factor in the elderly over time has been coined as ‘inflammaging’ by Claudio Franceschi [4]. There are several postulated mechanisms of inflammaging that could lead to increased susceptibility to CVD. First, an increase in senescent cells due to ageing could alter the secretion profile leading to more proinflammatory cytokines. This phenomenon is not just restricted to the cardiovascular system, but is a global process that affects the whole body.

The PolSenior study conducted in 4979 Eastern Europeans aged ≥65 years, revealed that Interleukin-6 (IL-6) and C-reactive protein (CRP) levels were increased in an age-dependent manner in general. However, patients who did not have a history of CVD, type 2 diabetes, cancer or stroke exhibited a small but significantly lower level of these two markers (IL-6 and CRP) [55]. CRP levels in particular, appear to be critically linked to the development of cardiovascular diseases [56]. CRP in turn is known to be regulated by IL-6 and tumour necrosis alpha (TNF-α) [57]. A six-year study by a team at Johns Hopkins University revealed that large and sustained elevation of CRP levels are associated with a very high risk of CVD and mortality [58].

Tocotrienols have been well established as molecules that can effectively lower blood serum levels of CRP. In fact the CRP lowering activity of tocotrienols have been noted to be higher than that of tocopherol, by at least 20–50% more [59]. This positions tocotrienols as a compound that can combat ageing related CVD via the lowering of CRP levels.

More recently, the myocardium has been scrutinised further from an immunological point of view [60–63]. These new findings have cast a spotlight on the new and emerging field of immunocardiology. New and emerging data indicate that the myocardial and immunological ageing process are intimately linked and intertwined. A study published at the time of writing, by Ramos et al., presented interesting findings in this field. The team utilised targeted cell ablations and cell-transfer methods to demonstrate that CD4^+^ T cells from the heart-draining lymph node of aged mice mediates low levels of cardiac inflammation and mild functional impairment even in wild type mice, in the absence of clear tissue damage or infection [64]. It is unclear from this study, however, what pathways are specifically triggered and which factors are responsible for the detrimental effects seen. This continual presence of T cells within the myocardium presents a further consideration as a disease-modifying factor where cardiovascular disease is concerned, certainly in the elderly.

Tocotrienols can play a role in immunomodulation of T-cell activity by regulating gene expression. Wilankar et al., reported that genes such as concanavalin A and NF-κB were increased on short term exposure (4 h) but suppressed in murine lymphocytes upon long term treatment with γ-tocotrienol [65]. These two genes are associated with lymphocyte proliferation and immune activation respectively. This immunomodulation impact may potentially confer benefits in the myocardium by suppressing potentially pathogenic inflammatory activity from these immunologic cells.

### C) Indirect changes

The previous two sections dealt largely with the heart itself. However, this section and the following one will deal more broadly with the cardiovascular system.

### i) Dyslipidemia

Tocotrienols can alleviate hyperlipidemia and hypercholesterolemia, huge risk factors in cardiovascular disease (CVD) risks, through triglyceride (TG) and low density lipoprotein-cholesterol (LDL-C) reduction by up to 25% [66]. Work from our group previously showed the cardiovascular and metabolic health benefits of tocotrienol supplementation in tissue culture systems, rodent models as well as human studies [36, 38]. In these studies, tocotrienols reduced the production and transport of TGs in cells, rodents and human thereby reducing TG levels by 28%. Tocotrienols not only lower LDL-C and TG, but they are also anti-inflammatory and provide multiple points of health benefits to lower CVD risk. There has been speculation that TG synthesis is lowered by modulating lipogenic gene expression based on cellular studies [67]. However more in vivo and human studies are needed to confirm this observation.

The mechanism for cholesterol reduction, however, has been elucidated to a large degree. Tocotrienols can suppress the production of 3-hydroxy-3-methyl-glutaryl-coenzymeA (HMG-CoA reductase), the rate-limiting enzyme essential for cholesterol production [68]. Currently, statins remain the gold standard for the treatment of hypercholesterolemia. The mechanistic details of statin’s cholesterol lowering have been worked out in great details over the years [69].

Statins mimic the structure of HMG-CoA and poses as a competitive inhibitor, for the HMG-CoA reductase [70]. While statins are considered the gold standard for treating hypercholesterolemia, it isn’t without side effects. The incidence of these side effects is low however, but can nevertheless be devastating. The most common complaint is of muscle pain and soreness. In more rare and extreme cases, patients suffer rhabdomyolysis, a life-threatening damage to the muscles [71].

While, efficacious and effective, statins, at higher doses come with unfavourable side effects such as abnormal liver function tests, nerve dysfunction and muscle disease. Statins have been increasingly shown to pose greater risk to the elderly. A longitudinal study done in elderly women in Australia revealed that these women were at greater risk of diabetes when given high doses of statin [72]. Similarly, in South Korea, a population-cased cohort study done there also revealed that a small but significant risk exists of developing new onset diabetes mellitus (NODM) with extended use of statins in the elderly [73]. Thus, while one ailment is treated, the risk for another disease goes higher.

Tocotrienols however poses no risk in developing diabetes mellitus, in fact quite the opposite effect is seen. Due to its strong antioxidant activity, studies done by Ling et al., revealed that tocotrienols can alleviate oxidative stress in the beta-cells of the pancreas [74], thus improving insulin secretion. Excessive oxidative stress is one mechanism that impairs pancreatic beta-cell health, which in turn impairs glucose-stimulated insulin secretion (GSIS). Additionally, tocotrienols have also been shown to activate the expression of peroxisome proliferator activated receptor γ (PPARγ), a key gene crucial for improving insulin sensitivity [75]. Hence, tocotrienols can also ameliorate diabetes, a metabolic condition, whilst improving cardiovascular associated morbidities.

**Fig. 2:**
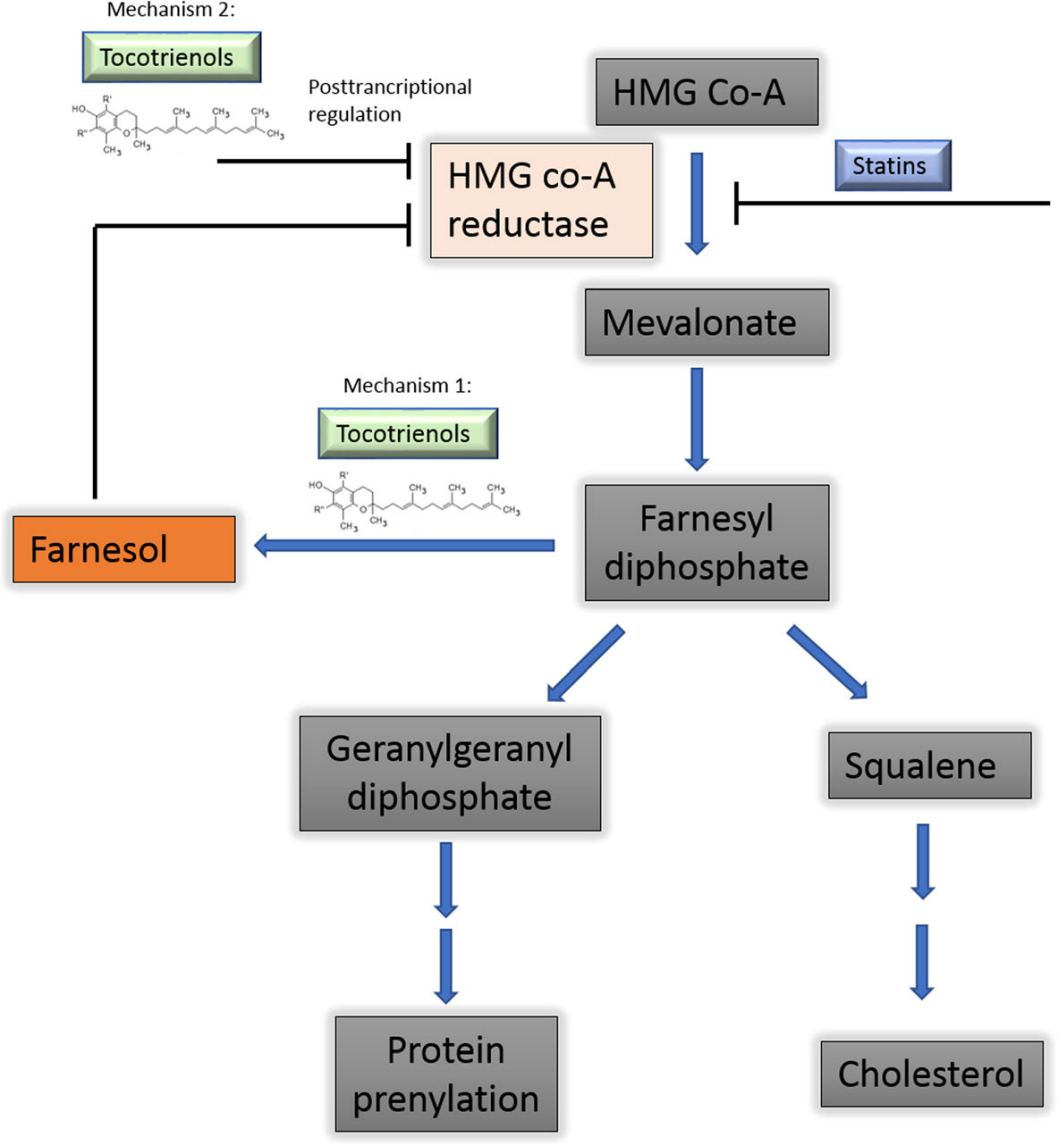
Tocotrienols lower cholesterol via 2 distinct mechanisms that ultimately operate on reducing the function of HMG-CoA reductase in catalysing the rate limiting step in cholesterol biosynthesis, as opposed to statins that have one mechanism of action, competitive inhibition by mimicking the native substrate HMG Co-A and binding the active site of HMG-CoA reductase. Mechanism 1 involves increasing the conversion of farnesyl diphosphate to farnesol, and this intermediate in turn accelerates the degradation of HMG Co-A reductase. Mechanism 2 involves a posttranscriptional means of regulation, by inhibiting the translation of HMG Co-A reductase mRNA. Both these mechanisms converge on HMG co-A reductase, the key rate-limiting enzymatic step in cholesterol biosynthesis

Statins inhibit cholesterol biosynthesis by acting as a competitive inhibitor of HMG-coA, the native substrate of the enzyme HMG-CoA reductase. Tocotrienols on the other hand inhibits cholesterol biosynthesis by two different and distinct mechanisms (Fig. 2):

1. Tocotrienols catalyse the dephosphorylation of farnesyl diphosphate to form farnesol due to its farnesyl tail (in comparison tocopherols have a phytyl tail that cannot perform this step) [76]. The resultant farnesol accelerates the degradation of HMG-CoA reductase [76], thus depriving the cholesterol biosynthesis pathway of the key rate-limiting enzyme.
2. Tocotrienols inhibits HMG-CoA reductase directly, posttranscriptionally, by blocking the translation of the mRNA [76, 77].

**Fig. 3:**
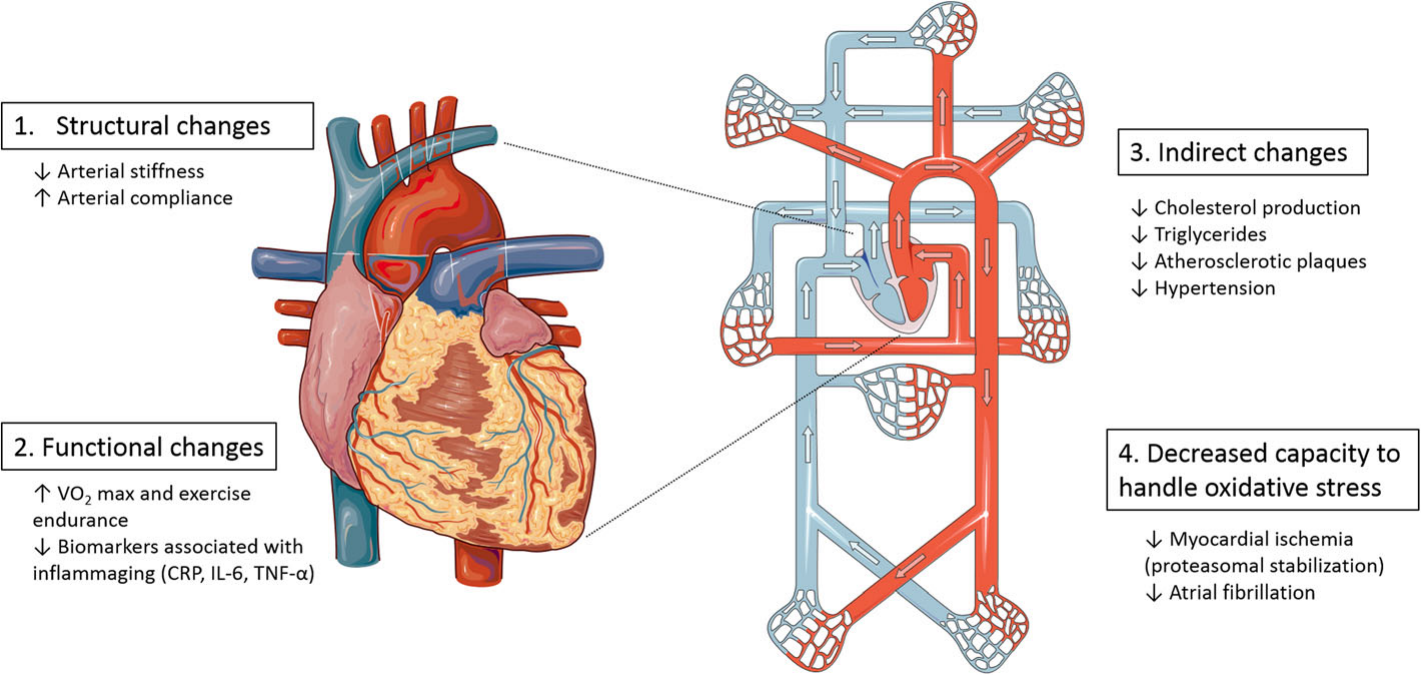
Summary of the potential cardiovascular benefits conferred by tocotrienol consumption in the elderly. Images are adapted from Servier Medical Art (http://smart.servier.com/)

A number of human trials have been conducted to investigate tocotrienols potential in lowering dyslipdemia, including hypercholesterolemia [66, 78–82], and all these studies have demonstrated significant improvement ameliorating the clinical parameters of dyslipidemia tested. There have been also studies that did not observe significant differences in improving dyslipidemia [83–85], including LDL-C and cholesterol parameters. We however note that in these studies, the subjects were not given a standardised diet or this information was not clear from the study, which could have led to confounding analysis of the resultant data. The inclusion criteria for some of these studies were also too broad, perhaps being another confounding factor. For example, Wahlqvist et al., employed a wide age range (25–75) [84], whereas it is well established that the basal metabolic rate of different age groups can affect the study outcome [86, 87].

There has been some interest in understanding the combinatorial effect of statin and tocotrienol. A study reported by Qureshi et al. demonstrated that co-treatment with lovastatin and tocotrienols led to a synergistic effect in improving the lipid profile of hypercholesterolemic subjects [88]. In other studies, combined treatment with tocotrienols and lovastatin have led to a decrease in cell proliferation in human cancer cell lines [89, 90]. Nevertheless, with the concerns posed by the intake of statins in the elderly, as discussed, there has been some caution with this approach.

Intriguingly, tocopherols, the more widely available form of Vitamin E, is known to antagonise the ability of tocotrienols to suppress HMG-CoA reductase. When tocopherol is added together with tocotrienols, cholesterol reduction is attenuated in cell culture [91], and animals [92, 93]. This antagonistic effect has not yet been unequivocally demonstrated in humans, possibly due to the complexities of interpreting data from the effects of more than one compound. However, most human studies employing α-tocopherol show no effect in reducing cholesterol or improving clinical dyslipidemia [94–96].

### Ii) lipid peroxidation and adhesion

Atherosclerosis is now well recognised as a chronic inflammatory disease. This disease is characterised by the accumulation of various species of lipids and inflammatory cells (which eventually form foam cells) in the arterial walls [97]. Endothelial injury and dysfunction are fundamental stimuli responsible for the formation of atherosclerotic plaque, and vascular wall inflammation is the key factor in the aetiology of atherosclerosis [98].

Oxidised LDL (ox-LDL) is a critical factor in the pathophysiology of atheromatous plaque, and this occurs due to lipid peroxidation by reactive oxygen species (ROS) (discussed more in Section D). Ox-LDL is preferentially taken up by macrophages via the scavenger receptors on these cells. This is done via non-LDL receptor pathway and as such it is unregulated and has no saturation point, allowing as much of these radicalised particles to enter the macrophages. Ultimately the macrophages convert to foam cells, which is a key turning point in the pathogenesis of atherosclerosis [25, 97–99]. Atherosclerosis-related clinical complications include coronary artery disease (CAD), stroke and other peripheral vascular diseases. CAD is one of the biggest killers in most developed and developing nations today [100].

Dietary tocotrienols have been shown to have a preventive role in the development of atherosclerosis in both animal models and humans [17]. α-tocotrienol, for an instance, has been shown to be 40 times more effective than α-tocopherol in providing protection to rat liver microsomal membranes from oxidative stress and damage [101]. α-tocotrienol was also found to show higher peroxyl radical scavenging potential than α-tocopherol in liposomal membranes [24]. More recently, however, δ-tocotrienol is becoming regarded as the homologue with strongest activity amongst tocotrienols where lipid peroxidation inhibition is concerned [102].

In humans, tocotrienols are reported to lower the stimulated endothelial cell expression of adhesion molecules (sVCAM-1, sICAM-1 AND e-selectin) by preventing the activation of NF-κB. By so doing, monocyte-endothelial cell adhesion is also decreased, and this correlation was seen to operate in a dose-dependent manner [103]. These findings have been also reproduced and expanded upon by other researchers [104, 105]. Similar to what was seen in the rodent studies earlier, δ-tocotrienol shown to be the most robust inhibitory effect on inhibiting the expression of sVCAM-1 in both these studies.

In recent times, and as alluded to when discussing dyslipidemia earlier, tocotrienols have been shown to be important in activating PPAR family of nuclear receptors, namely PPARα, PPARγ and PPARδ [75]. A diet rich in palm-derived TRF was found to reduce atherosclerosis development in ApoE^−/−^ mice through peroxisome proliferator activated receptor (PPAR) target gene liver X receptor alpha (LXRα) as well as other downstream target genes such as apolipoproteins and cholesterol transporters [106]. This suggests that another important aspect of tocotrienol activity in preventing atherosclerosis is by modulating PPAR activity. The mechanistic details as to how this is achieved is still a subject of intense research as at the time of writing.

In summary, tocotrienols may confer anti-atherosclerotic in a multi-pronged fashion, by not just being antioxidative and anti-inflammatory, but also in modulating the expression of important proteins are vital in the development of atherosclerosis, particularly in the elderly.

### Iii) hypertension

Arterial hypertension, or more commonly referred to as high ‘blood pressure’ (BP), is defined as systolic BP of greater than 140 mmHG or diastolic BP greater than 90 mmHG [107]. Ageing is linked to functional, structural and mechanical changes in arteries that closely resemble the vascular changes observed in the pathogenesis of hypertension [108]. There is now almost irrefutable evidence from a huge number of clinical trials that lowering hypertension can reduce the incidence of myocardial infarction [109].

A number of treatments exist in lowering systolic or diastolic BP. Most well-known of these are perhaps the ‘beta-blockers’, so-called because they block the beta-adrenergic receptors, thus leading to endothelium relaxation and lowering of BP [110]. Over the years, beta-blockers have proven to be efficacious in several other indications as well, most notably anxiety [111]. However, there have been a number of adverse events reported with the use of beta-blockers. Studies done with the elderly, in particular, report increased number of adverse events [112], and also more limited efficacy in addressing hypertension and cardiovascular-associated morbidities [113].

In addressing the above concerns, alternative and more recent medications have been used for the elderly. These include angiotensin receptor blockers (ARB), angiotensin converting enzyme (ACE) inhibitors and calcium channel blockers (CCB’s) [112, 114, 115]. Comparative studies done with ARB’s, ACE inhibitors and CCBs versus beta-blockers indeed show greater efficacy and reduced adverse events. [116–118] . For example, Kuti et al., reported reduced odds of developing new onset type 2 diabetes with CCBs compared to beta-blockers [119]. However, side effects such as dizziness and edema are also reported with some of these new medications [120].

Several studies in recent times have also reported the benefits of dietary antioxidants in reducing BP. Notable of these, is the ‘Dietary Approaches to Stop Hypertension (DASH) study [121], which is a multicentre, randomized, controlled-feeding trial where various combination of food items in lowering BP were studied. Diets rich in antioxidant Vitamin C (266 mg/day compared to 133 mg/day in the control group) was found to lower BP by 5.5/3.0 mmHG (systole/diastolic) in a subpopulation with moderately high BP and 11.5/3.0 mmHG in a subpopulation with clinical hypertension. The DASH study however doesn’t investigate the effect of a single nutrient, thus not allowing us to narrow down to the exact causative factor(s) of the observed improvement in hypertension. Since the DASH study however, more studies focussing on individual antioxidant nutrients have been conducted which includes tocotrienols [107].

Studies involving the role of tocotrienols in hypertension are currently limited to animal models, but the resulting data appear promising. Muharis et al., reported that palm-derived TRF may potentially improve vascular endothelial function in hypertension by improving endothelium relaxation in rat aortae [122]. Another study by Ali and Woodman, also done using palm-derived TRF, reported that TRF is better than pure tocotrienols at improving endothelium-dependent relaxation in rat aortae, suggesting that the combined effect of tocotrienols and α-tocopherol are both important in bringing about the improvement seen in the endothelium [23]. A follow-up study by the same team, using a similar setup as previously, but this time incorporating a high-fat diet, reported a similar improvement in endothelium-dependent relaxation, when given TRF [123]. Thus, palm-derived TRF, could provide a unique therapeutic opportunity for the elderly, by preventing the potential adverse effects seen with stronger pharmacological agents as described earlier. Ultimately, human studies will need to be conducted to confirm these beneficial effects in improving hypertension. However, these preclinical data do provide a strong basis to conduct further investigation in humans.

### D) Decreased capacity to handle oxidative stress – Mitochondrial dysfunction

Reactive oxygen species (ROS) and reactive nitrogen species (RNS) have been firmly established as both potentially harmful, but also potentially beneficial as signalling molecules in certain instances [124–127]. Both ROS and RNS are normally generated by tightly regulated enzymes such as nitric oxide synthase (NOS) and nicotinamide adenine dinucleotide phosphate (NADPH) oxidase. The mitochondrial electron transport chain is another source for the generation of ROS/RNS. The chronic overproduction of ROS/RNS, a phenomenon often linked with ageing [128], can be damaging to various parts of cell and tissue components, especially since it directly damages the important biomolecules key to sustaining life, such as lipid (lipid peroxidation), proteins and even DNA [126, 128, 129].

Harman proposed the free radical theory of ageing in the 1950s and this was later expanded in the 1970s to reflect the role of the mitochondria in the generation of ROS/RNS [128]. In a nutshell, this theory states that the ageing process is known to accelerate the overproduction of these species, leading to an increased oxidative burden that needs to be cleared by the body. The longer these ROS/RNS species occupy the system without being cleared, the higher the likelihood of them causing harm to the body. In the heart, the decline in function in the mitochondrial respiratory chain complexes, particularly complexes I and IV have been identified as one of the major reason for this [130].

This deterioration in mitochondrial energetics and function, leading to mitochondrial dysfunction, is gaining recognition as a major determinant in ageing-related cardiovascular disease [131]. A similar type of mitochondrial dysfunction is seen in radiation-induced heart disease (RIHD). In RIHD, exposure to radiation (such as in radiotherapy) leads to an increase in membrane permeability and subsequently impaired functionality of the respiratory chain complexes. One study by Sridharan et al., demonstrated the efficacy of TRF in ameliorating mitochondrial dysfunction by sustaining succinate-driven mitochondrial respiration. It is likely that these benefits could be conferred to the elderly afflicted with cardiac disease, particularly if the pathological state can be linked to mitochondrial dysfunction.

### i) Myocardial ischemia & heart failure

Ischemic heart disease (IHD) is a common form of cardiovascular disease that ultimately results in myocardial infarction. Myocardial ischemia results from a disruption in the coronary blood supply, and the majority of this is attributed to atherosclerosis. But, apart from atherosclerosis, oxidative stress in itself plays an important role in the pathogenesis of IHD [130, 132].

In post-ischemic myocardium, increased levels of ROS are also generated in the cardiomyocytes, endothelial cells, and infiltrating neutrophils. These in turn carve the path towards cellular dysfunction and necrosis. Substantial evidence exists to support the role of oxidative stress as one of the major aetiologies for myocardial injury [97, 127, 128, 131]. Besides, oxidative stress it thought to cause the occurrence of cardiac events after reperfusion therapy in acute coronary syndrome (ACS) [133]. Feng et al. demonstrated the correlation between increased oxidative stress marker plasma advanced oxidation protein products (AOPP) concentration and increased incidence of major cardiac events in patients treated with percutaneous coronary intervention for ST-segment elevation myocardial infarction during a six-month follow up [134]. In addition, Hokamaki et al. also showed that patients with high thioredoxin levels suffered from a more frequent recurrent angina attack as compared to patients with low thioredoxin levels after treatment of unstable angina [135]. Apart from that, studies have also revealed that there is a correlation among oxidative stress, ventricular remodelling and progressive dilatation leading to end-stage heart failure. Indeed, increased levels of oxidative stress biomarker such as malondialdehyde, lipid peroxidases, glutathione peroxidase, thioredoxin or superoxide dismutase have been shown to be associated with heart failure both acute and chronic conditions [136–138]. Additionally, these conditions are made worse by nitrate therapy, used widely in the treatment of both coronary artery disease and congestive heart failure. Apart from rapid development of nitrate tolerance, Munzel et al., reported that in vivo nitrate use can lead to the generation of superoxide anions from the endothelium [139]. Fan et al., conducted a clinical trial to investigate the effects of nitrate therapy and confirmed the significant increase of ROS/RNS in the elderly compared to younger patients [128].

The superior free radical scavenging activity demonstrated by tocotrienols, should lead to a considerable attenuation in oxidative stress-induced IHD as well as post-ischemic myocardial therapy. Studies conducted by a couple of groups have postulated that the cardioprotective value from tocotrienols may stem from their ability to stabilise proteasomes [140, 141]. Proteasomes become destabilised after ischemia [141, 142]. The ability to stabilise proteasomes after an ischemic episode allows a better balance between survival and apoptotic signals, improving myocardial health.

### Ii) atrial fibrillation

Atrial fibrillation (AF) is the most frequent postoperative complication after cardiac surgery afflicting the elderly. The prevalence rate of AF can range from 25% for coronary artery bypass surgery to 65% for in-valve replacement procedures. Postoperative AF after cardiac surgery not only doubles the morbidity rate but can increase mortality as well. Oxidative stress has been implicated in the pathogenesis of AF [143], and naturally antioxidant therapy has been suggested as an intervention strategy.

Interestingly, both Vitamin C and Vitamin E (tocopherols), have been investigated and proposed as a suitable prophylactic agent for addressing AF in numerous studies [143–146]. In particular, a meta-analysis conducted by Hemila and Suonsyrja found that of the 15 trials that used Vitamin C as a prophylactic treatment, AF risk was reduced by 27% on average, although the study also did report a huge heterogeneity in the way these 15 studies were conducted [145].

A recent study investigated if serum Vitamin E (tocopherol) level was related to AF recurrence in patients undergoing electrical cardioversion (EC) [147]. One hundred and forty-four consecutive patients who underwent successful EC were prospectively enrolled and followed for 3 months. It was indicated that low serum Vitamin E level was an independent predictor for AF recurrence.

Another study by Rodrigo and colleagues even postulated, based on their data, that Vitamin C therapy, in combination with omega-3 polyunsaturated fatty acid (PUFA), could become more efficacious with ageing when treating AF [144]. Given what is known about the potent antioxidant activity, compared to Vitamin C and tocopherols, tocotrienols could potentially further reduce the risk in AF, and presents a unique and novel method for addressing postoperative AF.

To our knowledge, no study examining the effect of tocotrienols have been conducted to date. Given these interesting findings with regards to antioxidant therapy, it is likely that tocotrienols could emerge as a potential candidate for treating AF based on its antioxidative profile.

## Conclusion

Whilst numerous reviews extolling the benefits of tocotrienols in cardiovascular diseases have been written, we chose to focus our review on a more specific subset of cardiovascular disease, namely ageing-related cardiovascular diseases (Fig. 3). With the advent of modern medicine and the technologies that accompany it, we have managed to prolong our lifespans and age gracefully.

Whilst prolonging our mortality has been a great achievement for mankind in medical science, we still grapple with some of the consequences of ageing, and cardiovascular-associated morbidities are one of them [148]. The ageing process is also one that is mired with increased oxidative stresses and inflammation. Pharmacological agents in the form of synthetic entities, exist to treat these ailments or its symptoms, but there can often be harsh side effects, which are often made worse in the elderly. As a result, there is an increasing interest in turning to compounds that exist naturally in nature and to harness their potential for clinical interventional strategies.

Apart from tocotrienols, two other compounds that are gaining interest in this field is curcumin and resveratrol. These two examples fall under the family of polyphenols. Significant research has demonstrated the antioxidant and anti-inflammatory effects of curcumin [149] and resveratrol [150], in the treatment of cardiovascular diseases The polyphenol, curcumin, is the active component of turmeric, a common Indian spice, derived from the rhizome of the *Curcuma longa* plant. Curcumin is the most abundant constituent of turmeric; comprising approximately 2%–5% of the compound [151]. Curcumin has been particularly noted in its ability to suppress inflammation by regulating multiple cytokines such as beta-site APP-cleaving enzyme (BACE-1), C-reactive protein (CRP) and MMPs, TNFα and NF-κB [151]. It has additionally been suggested that curcumin may modulate hypertrophy in the aging heart by inhibiting the Adenoviral transcription co-activator, p30 [149]. Interestingly, α-tocopherol levels were found to be greater with curcumin supplementation indicating the enhancement of endogenous antioxidant mechanisms. Despite the strong evidence, curcumin suffers from a poor bioavailability as evidenced in clinical trials [152]. Resveratrol has been extensively researched for its ability to modulate determinants that are linked with increased cardiovascular risk, in particular by stimulating the activity of sirtuins, particularly SIRT1, a histone deacetylase. Resveratrol is also a COX1 inhibitor which translates to reduced endothelial inflammation [153]. Elevated levels of resveratrol mimic caloric restriction in older adults, and the cardiovascular benefits of these are well documented. However, there is also evidence to show that in certain circumstances polyphenols such as resveratrol can bind and form complexes with proteins and minerals, thus impairing its efficacy. Thus, it is worth considering other potential alternatives such as tocotrienols.

Tocotrienols have been receiving a great deal of attention over the last 3 decades, especially with the discovery of its potential to ameliorate a wide range of disease conditions, due its superior antioxidant and anti-inflammatory activity.

It might perhaps be surprising as to how such a potent compound with enormous potential has not been hugely exploited yet. As mentioned earlier, apart from being a recent discovery, tocotrienols present a challenge in its pharmacokinetic and pharmacodynamic profile. From Fig. 1, one could see why this is the case. Tocotrienols present 3 C-C double bonds in their phytyl tail, as opposed to tocopherols, that have completely saturated C-C bonds. This makes tocotrienols much more hydrophobic or lipophilic, than its counterpart tocopherol, and subsequently a challenge to deliver orally. Nevertheless, there has been noteworthy progress in these areas. Self-emulsifying drug delivery systems (SEDDS), which employ a clever composition of isotropic mixtures of oils, surfactants, solvents and co-solvents/surfactants is one major strategy employed in devising formulations in order to improve the oral absorption of highly lipophilic natural compounds [154–156].

There have also been concerns about the potential side effect that tocotrienols could present in humans. It has been shown that tocotrienols exhibit lower IC_50_ concentration than tocopherols for the same concentration. However, up to date, there have been no serious adverse events reported in humans in all the human trials reported so far in the literature or on Clinicaltrials.gov. One study by Springett et al., performed dose-escalation studies of up to 3200 mg of pure delta-tocotrienol [157], which is one of the highest used in the literature to our knowledge. At this level, 2 patients reported diarrhoea. However, 3.2 g per day is not a feasible amount to consume daily from a practical point of view.

There have also been some concerns raised in recent times, if a highly potent anti-inflammatory compound might be so potent as to suppress even the normal immune functions of the body. After all, inflammation is a natural response of the human body to combat infection and other potentially injurious agents to our system. The possible side effects of anti-inflammatory agents to the host defence in a recent review in Cell [158]. Most of what was discussed however involved pharmaceutical drug compounds. Most, if not all, of these drug compounds are synthesised in the laboratory whereas nutraceutical compounds such as tocotrienols come from nature and have co-evolved with humans and other organisms over the course of time [159, 160]. Humans have in fact been consuming tocopherols and tocotrienols (albeit as a mixture with other nutrients and at much lower levels) from food products such as barley and rice for thousands of years. Thus, the human body have had a long time to adapt to tocotrienols and it is unlikely any major compromise would occur in the host defence. While no major side effects have been described with the use of tocotrienols, as mentioned above, future trials may be warranted to consider the immune function of individuals consuming tocotrienols as an enriched fraction however. Thus, based on the evidence available to date, tocotrienols are a safe and potential candidate in improving cardiovascular health, especially for the elderly, who can be more susceptible more aggressive pharmaceutical interventions. In addition to this, there are also other health benefits related to reducing oxidative stress and pathological inflammation which plays a role in providing holistic health benefits for the elderly.

## Abbreviations

ACE: Angiotensin converting enzyme
ACS: Acute coronary syndrome
AF: Atrial fibrillation
AOPP: Advanced oxidation protein products
ARB: Angiotensin receptor blocker
CCB: Calcium channel blocker
CRP: C-reactive protein
CVD: Cardiovascular disease
EC: Electrical cardioversion
IHD: Ischemic heart disease GRAS: Generally Regarded as Safe
LVH: Left ventricular hypertrophy
NOS: Nitric oxide synthase
PUFA: Polyunsaturated fatty acids
RNS: Reactive nitrogen species
ROS: Reactive oxygen species
SEDDS: Self-emulsifying drug delivery system
TG: Triglyceride
TRF: Tocotrienol-rich fraction
WHO: World Health Organisation
